# *Taenia saginata* prevalence in cattle slaughtered at low throughput abattoirs in South Africa

**DOI:** 10.4102/ojvr.v91i1.2157

**Published:** 2024-12-04

**Authors:** Mbali P. Dube, Charles Byaruhanga, Pierre Dorny, Veronique Dermauw, Daniel N. Qekwana

**Affiliations:** 1 Department of Veterinary Tropical Diseases, Faculty of Veterinary Science, University of Pretoria, Pretoria, South Africa; 2 Department of Biomedical Sciences, Institute of Tropical Medicine, Antwerp, Belgium; 3 Department of Paraclinical Sciences, Faculty of Veterinary Science, University of Pretoria, Pretoria, South Africa

**Keywords:** abattoir, bovine, cysticercosis, Gauteng, meat inspection, *Taenia*, tapeworm, South Africa

## Abstract

Meat inspection is the routine method used to identify cattle infected with *Taenia saginata*; however, the sensitivity of this method is low. We investigated the prevalence of *T. saginata* infection in cattle slaughtered in low throughput abattoirs (LTs) in Gauteng province, South Africa, based on meat inspection and serology. A total of 188 cattle carcasses from three abattoirs underwent meat inspection for the presence of *T. saginata* cysticerci, while serum was tested for the occurrence of antigens using antigen enzyme-linked immunosorbent assay (Ag-ELISA). Polymerase chain reaction (PCR)-sequencing was performed to confirm *T. saginata* cysts identified during enhanced inspection and incision of 10 randomly selected hearts. Apparent and true prevalence were calculated, and a logistic regression model was fit to evaluate associations between abattoir, sex, animal origin, age and *T. saginata* serological status. Out of the 188 carcasses, no positive cases (0%) were identified during routine meat inspection, yet three cysticerci-like lesions were identified from three hearts following additional incisions, of which one was confirmed as *T. saginata*. Fifty-four of the sampled cattle carcasses tested positive using Ag-ELISA (apparent and true prevalence: 29.0%, 71.8%). Feedlot cattle were less likely (odds ratios [OR]: 0.33, *p* = 0.043) to have a positive serological test result for *T. saginata* compared to non-feedlot cattle, and the odds of a positive result differed between abattoirs (*p* < 0.05).

**Contribution:**

Our results confirmed the low sensitivity of routine meat inspection in LTs, which may pose a public health risk, and therefore other diagnostic methods need to be included in the surveillance system for *T. saginata*.

## Introduction

*Taenia saginata,* a zoonotic parasite, causes cysticercosis in cattle and taeniosis in humans (McFadden et al. 2011; Phiri et al. 2002; Teklemariam & Debash 2015). Humans act as the definitive host, while cattle are the intermediate host, harbouring the larval stage (metacestode).

Cattle become infected with *T. saginata* when grazing on pasture or drinking from a water source contaminated with *T. saginata* eggs that are shed by a tapeworm carrier (WHO/FAO/OIE 2005). Humans are infected after consuming undercooked or raw beef contaminated with viable cysticerci of the parasite. Following ingestion, the cysticercus develops into a mature tapeworm within the small intestine of humans (Bucur et al. 2019; Lightowlers, Rolfe & Gauci 1996). *Taenia saginata* infections in humans are usually mild with non-specific clinical signs including anal itching, nausea, weight loss, diarrhoea, loss of appetite and mild abdominal discomfort (Dorbek-Kolin et al. 2018; Tsotetsi-Khambule et al. 2018).

In South Africa, *T. saginata* cysticercosis was reported as one of the most significant diseases of cattle, with losses mainly attributed to condemnation or freezing of infected carcasses (Dzoma et al. 2011; Qekwana et al. 2016; Tsotetsi-Khambule et al. 2018). Cattle grazing on communal lands are at a higher risk of infection than those in commercial herds, as they are more likely to be in contact with human faeces (Dzoma et al. 2011; Qekwana et al. 2016; Sungirai, Masaka & Mbiba 2014; Tsotetsi-Khambule et al. 2018). Furthermore, lower infection rates are usually detected in cattle slaughtered in low throughput abattoirs (LTs), slaughtering up to 20 cattle daily, than in those slaughtered in high throughput abattoirs (HTs) (Qekwana et al. 2016), the latter classified based on the capacity and nature of handling facilities (Department of Agriculture Forestry and Fisheries and National Department of Agriculture [DAFF & NDA] 2004). The relatively low detection rate in LTs can be because of the limited skills of meat inspectors working there with regards to the identification of *T. saginata* infected cattle (Qekwana et al. 2016).

Meat inspection is presently the sole routine method applied for the detection of *T. saginata* in South Africa (DAFF & NDA 2004); however, this method has lower sensitivity than serological tests (Abuseir et al. 2007; Dorny et al. 2000; Eichenberger, Stephan & Deplazes 2011; Jansen et al. 2018). A retrospective study involving meat inspection data (2010–2013) from cattle carcasses in 26 abattoirs (LTs and HTs) within Gauteng province of South Africa, revealed a total *T. saginata* detection level of only 0.7% (Qekwana et al. 2016). The low detection rate by meat inspection may make it difficult to estimate the actual risk of cysticercosis among cattle destined for slaughter, and therefore other approaches need to be integrated in the effective control of cysticercosis and taeniosis. In contrast, Tsotetsi-Khambule et al. (2017) demonstrated a cysticercosis seroprevalence of 15% from various localities in Gauteng province using the HP10 Ag-ELISA. The B158/B60 Ag-ELISA, which detects viable cysts, was revealed to have a much higher sensitivity (40%) and specificity (100%) in animals harbouring viable cysts as compared to meat inspection (Jansen et al. 2017). To our knowledge, there are no published reports that compare the efficacy of routine meat inspection and serological tests for the detection of *T. saginata* infection in LTs in South Africa. This study therefore assessed the prevalence of *T. saginata* infection in cattle slaughtered in LTs abattoirs by meat inspection and serological testing using B158/B60 Ag-ELISA and compared the efficacy of routine meat inspection with immunological testing in the identification of infected animals.

## Methods

### Study area

**FIGURE 1:**
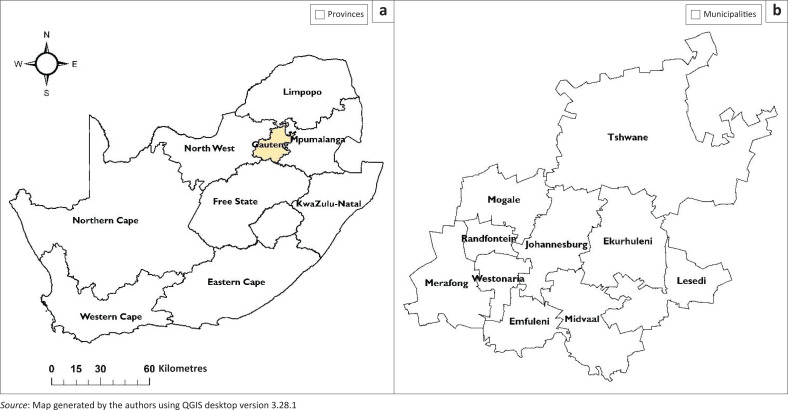
Map of South Africa showing the location of Gauteng province and its municipalities, where the study was conducted. (a) Provinces; (b) municipalities.

This study was conducted in Gauteng province (Figure 1) from May 2019 through October 2019. Gauteng is smaller than the other eight provinces in South Africa, occupying only 1.4% (18 178 km^2^) of the land area. However, it has the highest population of 14.6 million people. Gauteng is landlocked between four provinces, Free State to the south, North West to the west, Limpopo to the north and Mpumalanga to the east. It is divided administratively into metropolitan municipalities (*n* = 3), namely City of Johannesburg, City of Tshwane, Ekurhuleni and district municipalities (*n* = 2), West Rand and Sedibeng (Dyson 2009). South Africa is home to 13 934 125 head of cattle and 2 787 674 of these animals are slaughtered annually for human consumption. Gauteng province contributes 16.3% (*n* = 453 685) of the total 2 787 674 cattle slaughtered in South Africa annually (Statistic South Africa 2016).

### Study population and sampling approach

The study population was composed of cattle slaughtered in LTs in Gauteng. In 2018, there were eight registered LTs in Gauteng. At the time of the study, four of the abattoirs were not operational and the management of another abattoir did not consent to participate. Therefore, three abattoirs (A, B and C) participated in the study. Each abattoir was visited three times for sample collection. During each visit, all cattle presented for slaughter were included in the study. The required minimum sample size (*n*) was estimated using the following formula:

$$n=\frac{z_{1-\alpha /2}^{2}P(1-P)}{d^{2}}$$ [Eqn 1]

(Lemeshow et al. 1990), where *z*_1 – α/2_ is the *z*-value for a 95% confidence level, *P*, the expected prevalence and *d*, the precision (0.05). In this study, we used an expected seroprevalence of 15%, previously reported in Gauteng abattoirs (Tsotetsi-Khambule et al. 2017), resulting in an envisaged sample size of 196. A total of 188 head of cattle were slaughtered during our study trips.

### Sample collection

From each abattoir and each sample animal, we recorded this information: identification number, farm type (feedlot or non-feedlot), age (using dentition) and sex. Blood samples were collected into serum tubes from each animal during exsanguination. Then, routine meat inspection was performed by the meat inspector in each abattoir, as described in the *Meat Safety Act 40 of 2000* (DAFF & NDA 2004), on the same animals from which blood samples were collected. Briefly, inspection of cattle carcasses was performed through observation, palpation and incision. Hindquarters and forequarters were inspected by making incisions into the muscular tissue, while the inspection of the head was done by making multiple incisions into the external and internal masseters. The inspection of the tongue was done through palpation. Offals were visually inspected, and three incisions were made into the heart muscles.

The results of the routine meat inspection, in particular the number, location and development stage of cysticerci were recorded for each animal. Any recovered cysts presumed to be that of a parasite were obtained and stored in 80% ethanol for further analysis. Cysts were placed in ethanol, and together with blood samples, stored at 4 °C, transported to the DVTD laboratory in the University of Pretoria. Samples were centrifuged at 1200 g for 15 min, and serum was extracted and stored at –20 °C until analysis.

During one of the visits to abattoir B, we collected hearts of 10 of the 20 cattle slaughtered on that day, to enable enhanced meat inspection in our laboratory by making additional incisions, and to confirm identification of *T. saginata* cysticerci using PCR-restriction fragment length polymorphism (RFLP) and deoxyribonucleic acid (DNA) sequencing. The 10 cattle were chosen by systematic sampling during carcass inspection in the abattoir. A sampling interval of 2 (10/20) was determined beforehand and the starting point was determined randomly from the numbers 1 to 20. Only 10 hearts were available for us to examine from abattoir B, and we were unable to collect hearts from other abattoirs because of non-consent from the respective management. The heart is the preferred predilection site for the diagnosis of cysticerci in cattle because of higher density of cysts and frequency of infection compared to other organs (Scandrett et al. 2009). Heart samples were transported on ice to the Pathology Section of the Faculty of Veterinary Science, University of Pretoria.

On arrival, collected hearts were inspected by making multiple lengthwise incisions (up to 10) into the muscles, from the base to the apex, followed by observation of cut surfaces for the occurrence of *T. saginata* cysts. *Taenia saginata* cysticerci were classified as viable, degenerated or calcified. Viable cysticerci are translucent, while degenerated cysticerci are soft, yet have lost their translucent character, and calcified cysticerci are hard and non-transparent. Three cysts presumed to be parasitic material were obtained from three heart samples and stored in 80% ethanol for further analysis.

### Detection of *T. saginata* DNA from cysticerci

The recovered cysts were analysed using a semi-nested PCR coupled with RFLP as previously described (Geysen et al. 2007), to confirm the presence of *T. saginata*. Each cyst was squashed and homogenised followed by extraction of DNA using the PureLink^TM^ Genomic DNA Mini Kit (ThermoScientific^TM^, LTC Tech South Africa [Pty] Ltd., Randburg, South Africa), following the manufacturer’s instructions. The DNA was stored at –20 °C until further analysis. The *T. saginata* mitochondrial 12S rDNA gene was amplified using primers TaenF (5′- GTT TGC CAC CTC GAT GTT GAC T -3′) and ITM TnR (5′ CTC AAT AAT AAT CGA GGG TGA CGG-3′) in a primary PCR (900 base pairs [bp]), followed by amplification using primers nTAE (5′- CGT GAG CCA GGT CGG TTC TTA T -3′) and ITM TnR in a secondary PCR, 798 bp (Geysen et al. 2007). The primary PCR mixture contained 5 μL of genomic DNA, 0.4 μM of each primer, 1× Phusion^TM^ Flash High-Fidelity PCR Master Mix (Thermo Scientific^TM^, Randburg, South Africa) and PCR grade water to a total volume of 25 μL. The thermocycling conditions were 98 °C for 10 s for initial denaturation, followed by 40 cycles at 98 °C for 45 s, annealing at 57 °C for 45 s, and 72 °C for 1 min and a final extension at 72 °C for 10 min. The secondary thermocycling conditions were as in the first PCR except that 30 cycles of amplification were conducted.

Restriction digestion of 6 μL of the secondary PCR products was conducted in 15-μL reaction mixtures containing restriction enzymes *Ddel* (3U, 10 000 U/mL); *Hinfl* (3U, 10 000 U/mL) and *Hpal* (3U, 5000 U/mL (New England BioLabs^®^ Inc., Ipswich, MA, United States), Cut Smart Buffer 1× (final concentration) and nuclease-free water (6.3 μL). The PCR and RFLP amplicons were visualised using electrophoresis on 3% agarose gels that were stained with ethidium bromide. The positive controls were *T. saginata* and *T. hydatigena* DNA kindly provided by the Institute of Tropical Medicine, Antwerp, Belgium, while the negative control was a reaction with nuclease-free water as template.

### Sequence and phylogenetic analyses

The 12S rDNA secondary PCR amplicons from sample R6 (with clear bands) were purified using the QIAquick PCR Purification Kit (QIAGEN, Hilden, Germany) and this was followed by sequencing at Inqaba Biotechnical Industries [Pty] Ltd (Pretoria, South Africa) with primers nTAE and ITM TnR. The identity of the new sequence was compared with that of the previously published *T. saginata* sequences using the Basic Local Alignment Search Tool (BLAST) (http://blast.ncbi.nlm.nih.gov/Blast.cgi). The sequence was aligned with published homologous sequences using Multiple Alignment using Fast Fourier Transform (MAFFT) version 7 (Katoh & Standley 2013), followed by comparison of genetic distances and phylogenetic analysis using Molecular Evolutionary Genetics Analysis (MEGA) software version 11.0 (Tamura, Stecher & Kumar 2021).

### Ag-ELISA for detection of *T. saginata* circulatory antigens

The serum was tested for circulating cysticercus antigens using the commercial B158/B60 Antigen-ELISA Kit (apDia, Turnhout, Belgium). The cut-off value was computed based on the optical density estimates of four negative samples using the Students t-test variation at a probability level of *p* = 0.001. The optical density of each test serum sample was compared with the calculated cut-off value to determine the result of the test (Dorny et al. 2000). The B158/B60 Ag-ELISA used in this study detects viable cysts, with a 40% sensitivity and 100% specificity (Jansen et al. 2017).

### Data management and data analysis

The data were entered in a Microsoft Excel sheet. Then, the proportion of seropositive samples and their 95% confidence intervals were calculated. The true prevalence of *T. saginata* was calculated based on the apparent prevalence (AP), taking into consideration the specificity (*Sp* = 100%) and sensitivity (*Se* = 40%) of the Ag-ELISA test used, as follows (Greiner & Gardner 2000):

True prevalence=[AP+Sp−1]/[Sp+Se−1] [Eqn 2]

The association between Ag-ELISA positivity on the one hand and sex, age and farm origin on the other hand was assessed using a logistic regression model. In the initial step, univariable logistic regression models were performed to assess the association between the predictor variables abattoir, sex, age, the farm of origin and *T. saginata* serological status. Because of the sampling design, univariate models for the predictor variables sex, age and farm of origin all included abattoir as fixed effect as well. Potential predictor variables with *p* ≤ 0.050 in the univariable models were included in the multivariable logistic regression model. The significance of the explanatory variables in the multivariable model was assessed at *p* ≤ 0.050. Odds ratios (ORs) and their 95% confidence intervals were computed. The Hosmer–Lemeshow goodness-of-fit test was used to assess the fitness of the final model to the data. All statistical analyses were computed using the Statistical Analysis System (SAS) 9.4 software.

### Ethical considerations

Ethical clearance to conduct this study was obtained from the University of Pretoria Faculty of Veterinary Science Animal Ethics Committee (No. V071-18). This study was approved by the Gauteng Department of Agriculture, Land Reform and Rural Development (GDALRRD) (reference number 12/11/1/1/6—991). Consent was obtained from the abattoir managers for the collection of blood samples, cysts and hearts from the slaughtered cattle.

## Results

### Study population

Of the cattle carcasses tested (*n* = 188), 34.0% (*n* = 64) were processed in abattoir A, 28.7% (*n* = 54) in abattoir B and 37.2% (*n* = 70) in abattoir C. Most carcasses tested were from male cattle (62.2%, *n* = 117/188), less than 2 years of age (61.7%, *n* = 116/188) and originating from feedlots (66.5%, *n* = 125/188).

### Meat inspection, PCR-restriction fragment length polymorphism and sequence analysis for *T. saginata*

**FIGURE 2:**
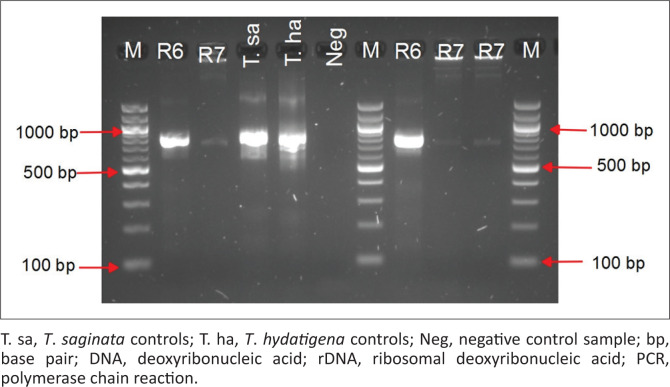
Agarose gel electrophoresis of semi-nested PCR amplicons (798 bp) of *Taenia saginata* 12S rDNA gene. The DNA was extracted from ‘likely to be cysts’ obtained from heart muscles of slaughtered cattle from an abattoir in Gauteng province, South Africa. Lanes M, 100 bp DNA size marker; Lanes R6 and R7, test samples.

**FIGURE 3:**
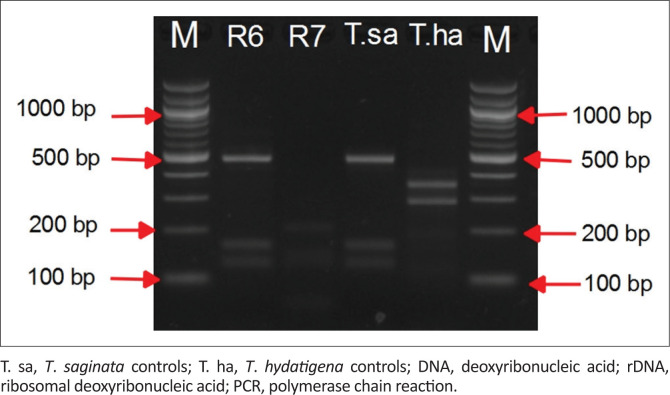
Agarose gel electrophoresis from restriction fragment length polymorphism of *Taenia saginata* mitochondrial 12S rDNA gene, achieved by digesting semi-nested PCR products of *T. saginata* using enzymes *Dde*1, *Hinfl*1 and *Hpa*1. Genomic DNA of test samples was obtained from ‘likely to be cysts’ from hearts of slaughtered cattle in Gauteng province, South Africa. M, 100 bp DNA size marker; R6 and R7, test samples; Electrophoresis was conducted in 3.0% agarose gels in TAE buffer.

Routine meat inspection did not reveal any positive cases of bovine cysticercosis (0%, 95% CI: 0.0–2.45). Fluid-filled lesions suspected to be cysticerci were collected from three different hearts out of the 10 hearts that underwent close inspection and further incisions. The cysts were classified as degenerating (*n* = 1) or calcified (*n* = 2). The corresponding carcasses from three head of cattle had been classified as negative for *T. saginata* during routine meat inspection at the abattoir. Of the three cysts tested, two (R6 and R7) yielded semi-nested PCR bands (Figure 2) that were identical to that of *T. saginata DNA* (~ 798bp). One cyst (R6) was confirmed to be of *T. saginata* upon PCR-RFLP with visible 471-bp, 165-bp and 128-bp bands (Figure 3) and by sequencing.

The new sequence has been deposited in GenBank under accession number MZ357054. On phylogenetic analysis, the sequence was grouped with *T. saginata* 12S ribosomal deoxyribonucleic acid (rDNA) sequences from cattle in Zimbabwe (accession number ON692929; 99.8% identity, 100% query cover), Iran (KF362126 and KC344680; 99.8% and 99.1% identity, respectively, 54% query cover) and China (PP391461; 99.9% identity, 100% query cover) (Online Appendix 1, Figure S1).

### ELISA detection of *T. saginata* antigens

Twenty-eight-point seven per cent (28.7%, 95% CI: 22.3, 35.2) of cattle carcasses were positive on Ag-ELISA (Table 1). Of these, 42.2% (95% Cl: 30.1, 54.3) were processed at abattoir A, 26.0% (95% Cl 14.2, 37.6) at abattoir B and 18.6% (95% Cl 9.5, 27.7) at abattoir C. The overall true seroprevalence of *T. saginata* antigens was 71.8% considering the performance of the Ag-ELISA test. Most positive animals were male cattle (31.6%, *n* = 37/117), up to 2 years of age (31.0%, *n* = 34/116) and originated from non-feedlots (31.8%, *n* = 20/63) (Table 1).

**TABLE 1:** Distribution of *Taenia saginata* infection based on B158/B60 Ag-ELISA among cattle from low throughput abattoirs in Gauteng, South Africa.

Variable	Category	Number of blood samples collected and tested	Number positive	%	95% CI
Abattoir	A	64	27	42.0	30.0, 54.0
	B	54	14	26.0	14.0, 38.0
	C	70	13	19.0	9.0, 28.0
Farm type	Feedlot	125	34	27.0	19.0, 35.0
	Non-feedlot	63	20	32.0	20.0, 43.0
Sex	Female	71	17	24.0	14.0, 34.0
	Male	117	37	32.0	23.0, 40.0
Age	≤ 2 years	116	34	31.0	23.0, 39.0
	> 2 years	72	20	28.0	17.0, 38.0

Note: Total number tested = 188.

95% CI, 95% confidence interval.

### Univariate analysis and multivariable logistic regression model

Abattoir (*p* = 0.011) and farm type (*p* = 0.010) were significantly associated with the serological test result in the univariable logistic regression models, while this was not the case for sex (*p* = 0.947) and age (*p* = 0.087) (Table 2). Abattoir and farm type were included in the multivariable logistic regression model (Table 3). In the final model, the odds for a positive serological test result for *T. saginata* were lower in cattle from abattoir B (OR: 0.34, *p* = 0.016), and abattoir C (OR: 0.14, *p* = 0.002) compared to cattle from abattoir A, whereas the odds were not significantly different for cattle carcasses processed at abattoir B as compared to those processed at abattoir C (*p* = 0.069). Feedlot cattle were less (OR: 0.33, *p* = 0.043) likely to test positive for *T. saginata* compared to non-feedlot cattle.

**TABLE 2:** The univariate analysis for the predictors of *Taenia saginata* antigen positivity in slaughter cattle in three abattoirs in Gauteng province, South Africa.

Variable	OR	95% CI	*p*
**Abattoir**	-	-	0.011
B	0.48	0.22, 1.05	-
C	0.31	0.14, 0.68	-
A	Ref.	-	-
**Sex**	-	-	0.947
Female	0.98	0.47, 2.02	-
Male	Ref.	-	-
**Farm type**	-	-	0.010
Feedlot	0.31	0.13, 0.76	-
Non-feedlot	Ref.	-	-
**Age**	-	-	0.087
≤ 2 years	0.49	0.22, 1.11	-
> 2 years	Ref.	-	-

95% CI, 95% confidence interval; OR, odds ratio; Ref., reference.

**TABLE 3:** Final multivariable logistic regression model for the predictors regarding *Taenia saginata* antigen positivity in slaughter cattle in Gauteng, South Africa.

Variable	OR	95% CI	*p*
**Abattoir**	-	-	-
B	0.34	0.14, 0.82	0.016
C	0.14	0.055, 0.40	0.002
A	Ref.	-	-
**Farm type**	-	-	0.043
Feedlot	0.33	0.11, 0.96	-
Non-feedlot	Ref.	-	-

OR, odds ratio; 95% CI, 95% confidence interval; Ref., reference.

## Discussion

The aim of the study was to investigate the prevalence of *T. saginata* in cattle slaughtered in LTs in Gauteng and to assess the efficacy of meat inspection compared to a serological test in the identification of *T. saginata* positive animals.

The negative result for *T. saginata* based on routine meat inspection in this study is not surprising as this method has been reported to have a very low sensitivity (Dupuy et al. 2014; Eichenberger et al. 2011; Geysen et al. 2007; Jansen et al. 2017; Marshall et al. 2016; Scandrett et al. 2009; Tsotetsi-Khambule et al. 2017). Our results agree with those of Dzoma et al. (2011) and Qekwana et al. (2016) in South Africa, who reported low proportions of *T. saginata* positive cases based on routine meat inspection among cattle in abattoirs in the North West (0.2%) and Gauteng provinces (0.7%) respectively. Elsewhere, some abattoirs in Spain also reported no *T. saginata* cases based on routine meat inspection (Allepuz et al. 2012). Meat inspection is not the only risk factor for the perpetuation of *T. saginata* transmission. Exposure to cattle is associated with herd management systems and sanitation practices in the community. Therefore, the likelihood of infection is expected to be higher in communal extensive systems where people practice open defecation. However, appropriate meat inspection lowers the risk of consumption of infected meat if the infected carcasses are condemned or treated, and identification rates of *T. saginata* positive carcasses in LTs relies on the motivation and skills of the meat inspector (Qekwana et al. 2016). Therefore, in carcasses with low infestation rates, viable cysts can be missed especially if insufficient incisions are made. Routine meat inspection should be continued in LTs as it can cheaply detect cysterici especially if infestation is high; however, there is need to enhance the skills of meat inspectors, especially in LTs, to minimise the risk of infected meat entering the food chain, more so in carcasses with relatively low infestation. If time and resources allow, the number of incisions on the predilection sites such heart, masseter, tongue and diaphragm can be increased as this is likely to improve the sensitivity of meat inspection in identifying *T. saginata* infected cattle (Jansen et al. 2017; Uys, Fosgate & Seguino 2023). The sensitivity and specificity of enhanced meat inspection (additional incisions in the heart) were found to be 2.87% and 100% respectively, following application of a Bayesian model to estimate prevalence and diagnostic test performance of bovine cysticercosis, by combining expert opinion with experimental data in the absence of a gold standard test (Jansen et al. 2018). However, extra incisions will impact the economic value of the carcasses (Uys et al. 2023) and may increase the risk of microbial cross-contamination. Abattoirs may need to strike a balance between economic benefits and public health protection by applying enhanced inspection on only a selection of carcasses on selected days in a particular season.

We detected circulating antigens of *T. saginata* in 28.7% of cattle using the B158/B60 Ag-ELISA. This was higher than the 23% and 15% previously reported in live cattle kept in rural areas in the Free State and Gauteng provinces respectively, using the monoclonal antibody (HP10) based Ag-ELISA (Tsotetsi-Khambule et al. 2017). Our figures are lower than the 34.9% prevalence reported from cattle slaughtered in abattoirs in Kenya using the HP10 Ag-ELISA (Onyango-Abuje et al. 1996). The difference in prevalence between the various studies can be explained by variation in management practices, which determine the rate of exposure of cattle to *T. saginata*, and therefore the level of infection detected in slaughtered animals.

Even in the same abattoir, daily detection rate may vary depending on the origin of the animals. The results of the current study suggest that *T. saginata* is common among cattle slaughtered in LTs in Gauteng, and that viable cysts, as detected by Ag-ELISA, are perhaps missed during routine meat inspection in the abattoirs. This is consistent with previous reports, for example Jansen et al. (2017), stating that Ag-ELISA can detect the presence of viable cysts not visible during meat inspection (Jansen et al. 2017). The B158/B60 Ag-ELISA is not species-specific, and cross-reactivity of *T. saginata* (cattle), *T. solium* (humans and pigs), *T. hydatigena* (cattle and pigs) and *T. ovis* (sheep) can occur. Therefore, interpretation of *T. saginata* results should take into consideration the geographical areas where *T. hydatigena* is prevalent in cattle (Nguyen et al. 2016). There are few published studies on *T. hydatigena* in cattle in Africa and none from South Africa. However, studies that have detected *T. hydatigena* in cattle show the prevalence was very low, between 0.2% and 5.2% (Nguyen et al. 2016). Therefore, false positivity because of cross-reactions is less important. Moreover, we did not observe *T. hydatigena* cysticerci in the inspected cattle. Overall, we thus consider that cross-reactions, if present, would account for a very small proportion of the Ag-ELISA positive results.

*Taenia saginata* DNA was detected during PCR-RFLP analysis of a cyst recovered during enhanced inspection of the heart of one animal; however, this animal was negative on routine meat inspection and Ag-ELISA. This can be explained by the fact that PCR detects the presence of parasite DNA in both degenerated and dead cysts (Geysen et al. 2007), whereas Ag-ELISA detects the presence of viable cysticerci (Brandt et al. 1992) and meat inspection is mainly able to identify heavily infested cases. The performance of these diagnostic tools should be investigated further under South African conditions, and to compare the performance of this serological test with meat inspection, by sampling in both LTs and HTs, and comparing the Ag-ELISA results with those of enhanced meat inspection so as to validate the performance of the test. Nonetheless, in the absence of serological testing or enhanced meat inspection at the abattoir, prevention of infection at the farm level is crucial. Therefore, the preventive measures such as good farming management practices, use of toilets and education and training of all stakeholders must be considered to mitigate transmission of *T. saginata* at the farm level (Nzeyimana et al. 2015; Rossi et al. 2017; Tsotetsi-Khambule et al. 2018). In addition, where resources are available, such as in medium to large-scale farms, serological testing (Ag-ELISA) can be applied in the surveillance for *T. saginata* at the farm level, to control transmission. If positive animals are identified on farms, these can be sent to abattoirs for slaughter and carcasses condemned or frozen following the regulations of the 2000 *Meat Safety Act 40* of the Republic of South Africa. The immunoassay may also be applied as a monitoring tool for *T. saginata* infections by the veterinary department at provincial or district levels. However, cross-reactivity between *T. saginata* and *T. hydatigena* antibodies can be a limitation if the latter parasite occurs in an area.

There were no significant associations between age, sex and seroprevalence of *T. saginata.* However, the proportion of cattle infected with *T. saginata* differed across abattoirs. Allepuz et al. (2012) in Spain also reported differences in the identification levels of *T. saginata* between abattoirs. As the infection is not acquired at the abattoir, cases identified in the abattoir could reflect poor livestock management and husbandry practices where the cattle are sourced (Onyango-Abuje et al. 1996). Moreover, in this study, cattle from non-feedlots compared to feedlot cattle were more likely to test positive for *T. saginata*, consistent with the high proportion of *T. saginata* reported in cattle from communal farms compared to commercial farms in Zimbabwe (Sungirai et al. 2014). We hypothesise that the higher seroprevalence of *T. saginata* in non-feedlot cattle could be attributed to the lack of good farming practices (Qekwana et al. 2016; Tsotetsi-Khambule et al. 2018) and the exposure to a high burden of *T. saginata* eggs (Gebrie & Engdaw 2015; Jenkins, Brown & Traub et al. 2013).

A limitation of this study is that only three of the eight LTs in Gauteng province were available or consented to participate in the study, and only 188 cattle (compared to the minimum required sample size of 196) were presented for slaughter during our field visits. This may under-represent the cattle population slaughtered in LT abattoirs; however, our findings (higher detection rate using Ag-ELISA than meat inspection) point to the fact that carcasses destined for consumption may pose a higher risk for taeniosis in humans than indicated from meat inspection, unless surveillance is enhanced for more effective detection of cases. Only three investigations have happened on taeniosis in humans in South Africa (Dermauw et al. 2018), and of these, only one was conducted in Gauteng province, with the finding of 15 cases between 2009 and 2010 based on microscopic examination of faeces (Ilze 2013). More studies are needed in human populations in the study area by both laboratory analysis and history assessments, the latter to identify the consumption practices of the patients and origin of the meat they consume.

Our study did not include high throughput abattoirs; perhaps a more comprehensive epidemiological evaluation of the difference in meat inspection efficacy between low throughput and high throughput abattoirs, coupled with more epidemiological information (e.g., farm management) will improve the significance of the future analysis with regards to the sensitivity of meat inspection in South Africa. Furthermore, we did not include data on management practices from the farms and/or herds of origin of the sampled cattle, because this was difficult to get from the abattoirs’ management. Only one study has investigated on-farm prevalence of *Taenia* species among cattle in South Africa (Tsotetsi-Nkambule et al. 2017); therefore, there is need for more investigations on occurrence under different production systems and management practices in the country. Nevertheless, our findings provide useful data to guide further research and control strategies.

## Conclusion

We detected the presence of *T. saginata* among slaughtered cattle in LTs in Gauteng province, South Africa, using serology. No cases were identified using routine meat inspection. The results confirm that meat inspection has a lower sensitivity in the detection of *T. saginata* cysticercosis compared to other methods. As the origin of the animals is an important factor in the level of *T. saginata*, farmers should be trained on the epidemiology of both bovine cysticercosis and taeniosis. There is also a need for more sensitisation of consumers regarding preparation of meat for consumption. PCR and immunoassays may be difficult to apply in abattoirs, but these should be considered in the overall surveillance system for *T. saginata*, to facilitate prevention and control of bovine cysticercosis and taeniosis in South Africa.
